# Is Inspiring Group Members an Effective Predictor of Social Dominance in Early Adolescence? Direct and Moderated Effects of Behavioral Strategies, Social Skills, and Gender on Resource Control and Popularity

**DOI:** 10.1007/s10964-018-0830-9

**Published:** 2018-03-13

**Authors:** Marjolijn M. Vermande, Patricia A. Gilholm, Albert H. A. Reijntjes, Dave J. Hessen, Elisabeth H. M. Sterck, Anne M. Overduin-de Vries

**Affiliations:** 1 Department of Child and Adolescent Studies, Utrecht University, Utrecht, The Netherlands; 2 School of Mathematical Sciences, Science and Engineering Faculty, Queensland University of Technology, Brisbane, QLD Australia; 3 Department of Methodology and Statistics, Utrecht University, Utrecht, The Netherlands; 4 Animal Science Department, Biomedical Primate Research Centre, Rijswijk, The Netherlands; 5 Department of Biology, Utrecht University, Utrecht, The Netherlands

**Keywords:** Social dominance, Resource control, Popularity, Resource control strategies, Inspirational behavior, Social skills

## Abstract

Dominance in the peer group is important for adolescents. Resource Control Theory posits that both coercive and prosocial (positively assertive) strategies are associated with dominance. Combining Resource Control Theory with Socioanalytic Theory on personality, we hypothesized that inspiring group members would be an additional effective strategy. This study examined whether the three behavioral strategies and two types of social skills (social competence and manipulation) predicted dominance (resource control and popularity). Participants were 619 Dutch adolescents (*M*_age_ = 13.1; 47% female) in the first grade of secondary school. They completed peer reports (behavioral strategies and dominance) and self-reports (social skills). Only inspirational and coercive strategies substantially predicted dominance. Main effects of social skills emerged. Moderation between strategies and social skills was only observed for girls (e.g., coercive strategy use was associated with more popularity for girls with higher levels of social manipulation skills). This study furthered our understanding of the predictors of dominance in adolescence by including inspirational behavior and examining prosocial and antisocial skills.

## Introduction

In humans and primates alike, social asymmetry (i.e., “competitive ability” or “the unequal distribution of status”) is a pervasive feature of groups (Sapolsky 2005). Individuals who attain high status generally experience benefits, whereas those with chronic low status experience profound disadvantages in various domains (Sapolsky 2005; Wilkinson and Pickett 2009). Although differential in-group status can already be observed in preschool, status is a key component of early adolescent peer relations (Brown and Larson 2009). Closson and Hymel (2016) argue that compared with youth of different ages, early adolescents prioritize status over many other domains, including maintaining friendships, engaging in romantic relationships, and achieving athletic or academic success.

The view that adolescence is a period of intensified peer interest and relationships has been generally acknowledged. The reason for the increased prominence of peers in this life stage lies in the consequences of a combination of maturational, psychological, and social changes, including puberty, autonomy, and social expansion (Brown 2011). Prior to adolescence, relatively little social interaction occurs between boys and girls. However, around puberty—usually regarded as the initiator of adolescence—the degree of other-sex contact increases substantially (Furman and Shaffer 1999). The transition to adolescence is also accompanied by a heightened drive toward autonomy (Zimmer-Gembeck and Collins 2003), which prompts adolescents to rely more extensively on peers. In addition, with the move to secondary school, adolescents encounter more peers, from a larger geographical area, than they did in elementary school. Because youngsters have to establish their social status amid the presence of peers mostly unfamiliar with each other, the struggle for high status usually increases, at least initially. This phenomenon has been observed for transitions in different grades (e.g., schools where the transition to secondary school is in Year 7 (roughly equivalent to grade 6), as well as schools where the transfer takes place in Year 8; Rigby 2007).

Different behavioral strategies can be used to achieve status. These strategies have been related to basic human motives or goals (Hawley 2003; Judge et al. 2009), of which “getting ahead” is easiest to link to status. In the present study, we draw on a recent social dominance approach (i.e., Hawley’s Resource Control Theory) and a contemporary theoretical view on personality (i.e., Hogan’s Socioanalytic Theory) to examine different behavioral strategies and the degree to which these strategies are associated with status.

### Resource Control Theory

According to evolutionary theorists (e.g., Buss 1996), people strive toward survival and reproduction in an environment that typically contains limited resources, which causes within-group competition. Because people differ in their ability and motivation to prevail in this competition, social dominance hierarchies emerge. Recent views define social dominance in terms of effective resource control (Charlesworth 1996; Hawley 2003; Pellegrini 2008). That is, social dominance is obtained by individuals who are especially effective at acquiring resources or “getting what they want.” Resources can be material (e.g., food, toys, best place in the school yard) or social (e.g., alliance partners, mates, social attention) (Hawley 2007). From an evolutionary perspective, resource control in children and adolescents may have immediate beneficial consequences, and these, in turn, may enable optimal growth, development (Willer and Cupach 2010), and health (Massey et al. 2014).

Traditionally, most scholars have defined social dominance in terms of aggression (i.e., force or force threat; Lewis 2002). That is, for ethologists, social dominance has been mainly construed as “getting ahead” (Hawley 2003, 2014). However, researchers increasingly recognized that aggression may not be the only way to obtain a strong position in one’s group and that cooperative, affiliative, and prosocial behaviors may also contribute to obtaining resource control (see Reijntjes et al. 2018, for a brief historical overview). Hawley’s (1999) influential Resource Control Theory is in line with this view. Resource Control Theory distinguishes between two broad classes of behavioral strategies, called *coercive* and *prosocial* resource control strategies (Hawley 1999, 2014). Coercive strategies refer to instrumental aggression (e.g., taking away, demanding, threatening) and they appear early in life: Toddlers and preschoolers predominantly apply coercive strategies. As verbal abilities and social skills to negotiate with their peers develop (around the age of 5), children may start to show prosocial strategies (Hawley 1999). Prosocial strategies pertain to positive, socially acceptable behaviors used to obtain resource control, including promising reciprocity and cooperation (e.g., swapping toys, promising friendship in exchange for something, “helping” other children who don't really need help). It should be noted that according to Resource Control Theory, prosocial strategies are self-serving rather than other-serving or altruistic (Hawley 2014, 2015). Nevertheless, prosocial strategies shape positive relationships with others, such as alliances and friendships. In other words, according to Resource Control Theory, prosocial strategies are motivated by “getting along” (Hawley 2003, 2014). Overall, Hawley and colleagues found coercive and prosocial strategies vary from being mildly to strongly positively correlated, depending on how they were measured (e.g., a strong correlation of *r* = 0.67 was found by observing preschoolers; Hawley 2007). They never observed negative correlations between the two types of strategies, suggesting a common function (i.e., obtaining resources; Hawley 2007).

Adolescents differ in the degree to which they apply coercive and prosocial strategies. Some adolescents, especially boys, are highly driven to “getting ahead” and apply mainly coercive strategies, whereas others, particularly girls, put “getting along” over “getting ahead” and predominantly use prosocial strategies (Hawley 2003). Importantly, according to Hawley and colleagues, both approaches are effective for obtaining above average resource control (Hawley 2007). However, this finding has not always been replicated by others (Olthof et al. 2011; Reijntjes et al. 2018). The study by Olthof et al. (2011), for example, reported that frequently and predominantly using prosocial strategies yielded only average resource control in pre-adolescents.

### Socioanalytic Theory

The basic motives of “getting along” (i.e., the need for companionship and social acceptance) and “getting ahead” (i.e., the need for status and control of resources) are central themes in Socioanalytic Theory on personality (Hogan 1983; Hogan and Blickle in press). They were derived from two important generalizations about human nature: That people always live in groups and that every human group has a status hierarchy. Hogan and Blickle (in press) present empirical support for these two motives underlying social interaction, using studies of different disciplines in both adults and youth. Socioanalytic Theory posits that people differ in their desire to get along and get ahead and the strategies they have developed to deal with these motives, as well as their success in attaining these goals. Social skill allows people to achieve their interpersonal goals “just as hand-eye coordination allows people to hit tennis balls accurately” (Hogan and Blickle in press, p. 19).

Importantly, Hogan and colleagues added a third motive to the familiar dual-motive approach. Based on results from social science and primate studies they infer that “People want their lives to be predictable, orderly, and sensible, and they fear chaos, randomness, and unpleasant surprises” (Hogan and Shelton 1998, p. 130). This motive is referred to as “finding meaning” (Hogan and Blickle in press). Finding meaning has been theoretically linked to leadership in organizations (Hogan and Blickle in press; Judge et al. 2009). Effective leadership would require not only getting ahead and getting along, but also “inspiring followers to strive toward a purpose that has meaning and the promise of fulfillment” (Judge et al. 2009, p. 861). As dominance and leadership are related (Cillessen et al. 2014; Waasdorp et al. 2013), *inspirational behavior*, as we call it, may also be associated with dominance. That is, adolescents who are able to inspire group members may be more proficient in getting what they want. Inspirational behavior, however, is not explicitly acknowledged by Resource Control Theory. We, therefore, aimed to examine inspirational behavior as a potential additional predictor of dominance in terms of resource control (i.e., the degree to which inspirational behavioral strategies were associated with resource control above and beyond the effects of coercive and prosocial strategies).

### Popularity as a Form of Social Dominance

As discussed above, recent evolutionary views define social dominance as differences in resource control among members of a group. Although the concept of popularity stems from a different (i.e., sociological) tradition, peer relations researchers increasingly construe popularity as a form of dominance (Hawley 2014; Pellegrini et al. 2011; Olthof et al. 2011; Rubin et al. 2015). Popularity is characterized by power, prestige, or visibility (Cillessen and Marks 2011) and is considered to be related to social dominance, as both resource control and popularity result from the strategic use of aggressive and prosocial behavior (Cillessen 2011). Yet, resource control and popularity are considered to be distinct manifestations of social dominance in adolescence (Dyches and Mayeux 2015). Therefore, we expected that the coercive, prosocial, and inspirational strategies may differentially affect popularity and resource control.

### Social Skills Related to Resource Control and Popularity

In line with Socioanalytic Theory, researchers often suggest that socially dominant youth have sophisticated social skills that allow them to execute behavioral strategies successfully (Hawley et al. 2007; Cillessen 2011). However, empirical research examining which social skills are related to social dominance, and how they are related, is limited (Wargo Aikens and Litwack 2011). The second aim of this study was to examine to what extent two different social skills, social competence and social manipulation, were predictors of dominance.

The effectiveness of social interaction is a defining feature of social competence (Wargo Aikens and Litwack 2011). Some studies have shown positive associations between social competence and dominance. Research on resource control has shown that a component of social information processing called sensitivity/attention to social cues (“I can tell when I’ve made others feel bad/good”) is correlated with prosocial resource control in late childhood and adolescence (Hawley 2003; Hawley et al. 2002). With respect to popularity, Andreou (2006) reported that components of social intelligence (i.e., social information processing, social skills, and social awareness) in school children were positively correlated with popularity, with correlations being somewhat stronger for girls than for boys. Puckett et al. (2008) examined the degree to which the effect of relational aggression on popularity was moderated by adolescents’ beliefs in their own ability to be social successful and efficacious in the peer group (i.e., social self-efficacy). Their results showed that social self-efficacy predicted popularity and also moderated (i.e., strengthened) the effect of relational aggression on popularity. In addition, pragmatic communication skills (i.e., the skills to choose the appropriate communicative acts given the context) were found to positively predict popularity in early adolescence. Pragmatic skills also moderated the association between prosocial behavior and popularity, such that those high on both prosocial behavior and pragmatic communication skills were most popular (Wolters et al. 2014). These results show that social competence may be directly related to resource control and popularity and may also moderate the relations between behavioral strategies and these two constructs.

As discussed above, past research has predominantly focused on the relation between *pro*social skills and dominance (Wargo Aikens and Litwack 2011). Social skills more negative or antisocial in nature, such as social manipulation (Riggio 1986), have consistently received less attention, but are likely to also be associated with dominance. Specifically, social manipulation is a general attitude or orientation which makes people “believe that in certain social situations it is necessary (and useful) to manipulate others or alter elements of the situation to affect the outcome of social encounters” (Riggio 1986, p. 651). It has been suggested that the ability to manipulate relationships is a skill that plays a role in the acquisition of popularity, and that some forms of aggression, for example, those used to gain a dominant position, could require social manipulation to be executed effectively (Cillessen 2011). Furthermore, adolescents who are most successful at obtaining resources have been referred to as “Machiavellians”, which in itself connotes social manipulation skills (Hawley 2003). Finally, although counter-intuitive, prosocial behavioral strategies may also require a degree of social manipulation as these behaviors are considered to be self-serving rather than other-serving. Although empirical evidence is limited, we expected that social manipulation would positively predict resource control and popularity and also moderate the association between the three behavioral strategies and both measures of dominance, such that the associations would be stronger for those who displayed higher levels of social manipulation.

### Gender Differences

Some gender differences have been found in regard to behavioral strategies and social skills during adolescence. As noted above, it has been reported that boys use more coercive strategies, whereas girls use more prosocial strategies to achieve and maintain peer status (Hawley et al. 2008). However, male and female adolescents are often found to be fairly equally represented among the most dominant members of a social group (Hawley et al. 2008). Girls are also reportedly more socially skilled than boys as indexed by general measures of prosocial skills (Merrell et al. 2011; Wargo Aikens and Litwack 2011). Therefore, it could be expected that for boys different behavioral strategies and social skills would be associated with dominance than for girls. This possibility was explored by investigating whether gender qualified the relations between the behavioral strategies, social skills, and social dominance.

## Present Study

By combining Resource Control Theory and Socioanalytic Theory, we conjectured that inspirational behavior may be a third behavioral strategy to gain dominance, above and beyond the effects of coercive and prosocial strategies. This study aimed to evaluate whether inspirational behavior was indeed an additional behavioral strategy yielding dominance—as indexed by both resource control and popularity—in early adolescent boys and girls. In addition, in line with Socioanalytic Theory and because empirical research linking social skills and dominance is scarce, we examined whether different social skills—social competence and social manipulation—were associated with dominance, either directly or by moderating the effects of the behavioral strategies. With regard to the first research question, we expected that inspirational behavior would be positively related to both prosocial and coercive strategies, and that inspirational behavior would explain a significant portion of variance in resource control and popularity. Regarding the second research question, we expected social competence and social manipulation to be positive predictors and, in addition, moderate the relations between the three behavioral strategies and both measures of social dominance. Specifically, we expected that there would be a stronger association between the strategies and dominance for those who scored higher on social competence and/or manipulation. Finally, we included gender as a potential moderator, to explore whether the direct or interaction effects differed for boys and girls.

## Method

### Participants

The original sample consisted of 732 young adolescents (*M*_age_ = 13.1 years, SD = 0.46; 53% males) in the first grade of secondary school. The students came from 27 classes located across the Netherlands. Due to technical problems, three classes did not provide data on any of the variables assessed with peer-ratings. As five of the seven study variables used peer-ratings that were standardized within classes, it was clear that imputation would be inappropriate, as there were no within-class data available to estimate these peer-ratings. Therefore, participants from these three classes were excluded. A comparison of the three excluded classes with the participating classes showed that there were no differences regarding age, gender, and social manipulation, but that the pupils in the excluded classes scored somewhat higher on social competence than those in the participating classes (*t* (693) = 6.78, *p* < 0.001). The effect size (Cohen’s *d* = 0.42) indicated that 84% of the two groups overlapped. An additional 34 students were absent and therefore had missing data on all self-report variables (but they still received ratings from classmates). No significant differences were found on peer-ratings between those who were absent and the remaining participants, except for inspirational behavior, *t* (651) = 2.69, *p* = 0.007, such that those who were present scored somewhat higher on inspirational behavior than those who were absent. However, this effect was small (*d* = 0.21; 92% of the two groups overlapped). All missing self-report values were then imputed using Multiple Imputation in SPSS. The analyses were performed for both the sample with complete cases and the sample with multiple imputation and no differences in the results emerged. Therefore, only the analyses performed on complete cases were reported. The final sample consisted of 619 adolescents (53% males). The age of the participants ranged between 11.6–14.8 years (*M* = 13.1, SD = 5.5), and 85.2% of the participants were native Dutch.

### Procedure

Paid research assistants approached schools by telephone using lists of secondary schools throughout the Netherlands. Individual schools were selected at random per province. The schools that showed interest then received further information. After obtaining definitive consent from the schools and classroom teachers, parents of the students received an information letter about the research, procedures, and data storage. They returned a signed form or contacted the school if they did not want their child to participate. On the day of data collection, students were given the option to decline or opt out of participating at any time, which none of the students did. Students completed an online questionnaire using Limesurvey (https://www.limesurvey.org/) that contained both self-report and peer-rating questions. All data kept by Limesurvey are stored on a secured university server, inside the university network. Questions were presented in a text format. Assistance by a research assistant, who was always present when the data were collected, was provided when needed. For the peer-rating questions, participants were presented with a list of all other participating students in their class. The names of the participating classmates were displayed in a random order, which was different for each question. Students could rate an unlimited number of peers (both boys and girls) within their class (Bukowski et al. 2012), or they could choose not to rate any peers for a particular question. The questionnaire was completed during class time in a computer-equipped classroom of the school under the supervision of the classroom teacher and a trained research assistant and took on average 45 min to complete. Afterwards, the schools received a brief report summarizing the main results per class.

### Measures

#### Behavioral strategies

Use of the three behavioral strategies was assessed via peer-ratings. The items tapping coercive and prosocial strategies were adapted from those used by Hawley, as per Olthof et al. (2011). Some of the original items confound the use of a particular strategy with the benefits that result from using that strategy (Olthof et al. 2011). For example, the items “Who makes others do what they want?” (Hawley et al. 2007), and “This child gets what s/he wants by making verbal threats or threats of aggression” (Hawley et al. 2007) refer not only to the adolescent’s behavior but also to the successful acquirement of social dominance as a result of using that behavior. In this study, in line with Olthof et al. (2011), care was taken to reword the items such that they would not imply that using the behavior would result in dominance (e.g., “Which children in your class *try* to get what they want by forcing others?”). For each type of behavior a total score was created by averaging the number of received ratings and then dividing by the number of nominating classmates. To correct for differences between classes in the way that adolescents report on each other’s behavior, as well as the non-normal distribution of the data, the scores were standardized and normalized within classes using the SPSS Rankit procedure (Olthof et al. 2011; Reijntjes et al. 2018; Salmivalli and Voeten 2004). The three behavioral strategies are described below.

##### Coercive strategies

Participants were presented with six items (e.g., “Which children in your class try to get what they want by forcing others?”; “… try to get others to do what they tell them to do, even if they don’t really want to?”). Participants nominated all the students in their class to whom the statements applied and then indicated whether the nominee displayed the behavior “sometimes” (scored as 1) or “often” (scored as 2). Cronbach’s alpha for this scale was 0.87.

##### Prosocial strategies

Five items were used (e.g., “Which children in your class act nicely in order to try to get what they want?”; “… promise to be friends in order to get what they want? For instance they say ‘‘I’ll be your best friend’’ ”). Participants nominated all the students in their class to whom the statements applied and then indicated whether the nominee displayed the behavior “sometimes” (scored as 1) or “often” (scored as 2). Cronbach’s alpha for this scale was 0.75.

##### Inspirational behavior

As yet no measure of inspirational behavior exists (this is also the case in leadership research based on Socioanalytic Theory; G. Blickle, personal communication, 1 November, 2016). Three items were created for this study to assess to what extent children used inspirational behavioral strategies. These items read as follows: “Which children in your class … (1) try to get others enthusiastic about something, so that they want to participate?, (2) try to convince others by explaining why their idea is a good idea?, and (3) try to persuade others to do something or not do something by giving advice or suggestions?”. Participants nominated all the students in their class to whom the statements applied and then indicated whether the nominee displayed the behavior “sometimes” (scored as 1) or “often” (scored as 2). Cronbach’s alpha for this scale was 0.87.

#### Social competence

Social competence was measured via self-report using the social competence subscale from the Social-Emotional Assets and Resilience Scale (SEARS; Merrell et al. 2011). This scale contains 12 items (e.g., being comfortable talking to others), which were scored on a four-point Likert-type scale ranging from 1 (“never”) to 4 (“always”). A social competence score was created by calculating the mean score of the items. Cronbach’s alpha for this scale was 0.86.

#### Social manipulation

Participants responded via self-report to three items assessing social manipulation adapted from the Social Skills Inventory (Riggio 1986). These items were (1) “Occasionally I lie to get something done”, (2) “If I really have to, I can “use” other people to get what I want”, and (3) “Sometimes I feel that the social rules that govern other people don’t really apply to me”. Items were scored on a five-point Likert- type scale ranging from 1 (“completely not true for me”) to 5 (“completely true for me”). A social manipulation score was created by calculating the mean score of the items. Cronbach’s alpha for this scale was 0.69. Because alpha was moderate and is known to be a lower bound to the reliability (Sijtsma 2009), we also computed McDonald’s omega, a greater lower bound to the reliability than alpha. The real reliability ≥ omega (Revelle and Zinbarg 2009). The estimate of omega for social manipulation was 0.75.

#### Resource control

The items to measure peer-reported resource control were adapted from those used by Hawley as per Olthof et al. (2011). Participants were presented with six items (e.g., “Which children in your class have the nicest items or the best place (when something is happening)?; “… usually are the center of attention in a group of children?”), and nominated the students in their class to whom the statements applied. They then indicated whether the nominee displayed the behavior “sometimes” (scored as 1) or “often” (scored as 2). Cronbach’s alpha for this scale was 0.87. A total resource control score was created by calculating the mean number of ratings received across the six items and then dividing by the number of nominating students in the class. These scores were then ranked and normalized per class using the SPSS Rankit Procedure.

#### Popularity

Participants nominated students in their class who they considered to be “most popular” and “least popular” (LaFontana and Cillessen 2002). Following the sociometric methods for assessing popularity in prior studies, these terms were not further explained to the participants (Cillessen and Marks 2011; LaFontana and Cillessen 2002). Perceived popularity scores were calculated within classrooms as the standardized difference between the standardized number of popular votes and the standardized number of unpopular votes the adolescent received (LaFontana and Cillessen 2002).

### Confirmatory Factor Analysis

Although conceptually different, both prosocial and inspirational strategies pertain to socially acceptable behavior. Therefore, a confirmatory factor analysis using mlm estimation (Satorra and Bentler 1994) was performed in Mplus on the rankit-transformed items to evaluate model fit and confirm the structure in the data. Support for the two-factor model would indicate that the items measuring inspirational and prosocial strategic behavior could be represented by two separate constructs, which would indicate that inspirational strategic behaviors are not equivalent to prosocial strategic behaviors. Several goodness-of-fit-indices were calculated: the relative/normed chi-square (*χ*^2^/df), the Comparative Fit Index (CFI), the Tucker-Lewis Index (TLI, also known as the Non-Normed Fit Index), the root mean square error of approximation (RMSEA), and the standardized root mean square residual (SRMR) (Hooper et al. 2008). Generally, acceptable threshold levels for the relative *χ*^2^ range from 2:1 to 3:1. CFI and TLI values ≥ 0.95, RMSEA values ≤ 0.07 and SRMR values ≤ 0.08 indicate good model fit (Hooper et al. 2008). The results confirmed the two-factor model (relative *χ*^2^ = 2.1; CFI = 0.97; TLI = 0.95; RMSEA = 0.04; SRMR = 0.03). In addition, the factor loadings confirmed the expected structure. Factor loadings of the five prosocial strategy items on the prosocial factor ranged from 0.23 to 0.54 (standard errors ranged from 0.034 to 0.040). Factor loadings of the three inspirational strategy items on the inspirational factor ranged from 0.44 to 0.66 (standard errors ranged from 0.040 to 0.041). The factors had a positive correlation of 0.51. A model with 1 factor resulted in a poor fit (relative *χ*^2^ = 25:1; CFI = 0.77; TLI = 0.68; RMSEA = 0.11; SRMR = 0.07). We concluded that prosocial and inspirational strategy use are clearly identifiable distinct constructs as measured by the items that are used in this study.

### Analytic Strategy

As the participants were nested within classes, we first considered a multi-level model. However, as the peer-rating variables were standardized per class, this reduced the intra-class correlations for resource control and popularity to zero. Therefore, a multi-level model was considered unnecessary and all analyses were conducted at the individual level in SPSS and R (https://www.r-project.org/). First, Pearson’s bivariate correlations and *t*-tests were calculated to determine the relationships between the study variables and gender differences. Subsequently, hierarchical regression analyses (with heteroskedasticity-consistent standard error estimators; see below) to assess the main and interaction effects of the study variables were conducted. Gender was coded as 0 for boys and 1 for girls. Social competence and social manipulation were centered prior to analysis and the computation of the interaction terms. The three dominance-oriented behaviors were already standardized, thus further standardization or centering was not necessary. All variance inflation factors (VIF) were below 10 (i.e., ranging from 1.06 to 4.14) and the tolerance statistics were above 0.20 (ranging from 0.24 to 0.95), indicating that multicollinearity was not an issue.

At the first step of the hierarchical regression analysis, coercive, prosocial, and inspirational behavioral strategies, social competence, social manipulation, and gender were entered to assess their main effects on resource control. In the second step, the six two-way interactions^1^ between the three behavioral strategies and the two social skills were added to examine whether the social skills moderated the association between the three behavioral strategies and resource control. In the third step, the five two-way interactions^2^ between (a) gender and the three behavioral strategies, and (b) gender and the two social skills were added. Finally, in the fourth step, all six three-way interactions^3^ between the behavioral strategies, social skills, and gender were entered, to establish whether the two-way interactions added in step two were moderated by gender. The four steps were repeated with popularity serving as the dependent variable. With respect to power, 10 to 20 participants for each independent variable are required (Keith 2006), or 230 to 460 participants for all the independent variables (main and interaction effects) of this study. The sample of 619 participants in the regression analyses thus provided sufficient power.

For the hierarchical regression analyses, we used robust standard errors. Using robust standard errors is advised, even if heteroskedasticity seems not to be an issue (Hayes and Cai 2007; Yamano 2009) as was the case in this study. The analyses were performed in R using the “sandwich” package (Zeileis 2006) with the Cribari-Neto’s heteroskedasticity-consistent standard error estimator (HC4; Hayes and Cai 2007). Partial eta-squared values were computed for each predictor in order to estimate the effect size. Partial eta-squared can be benchmarked against Cohen’s (1969) suggested criteria of small (0.0099), medium (0.0588), and large (0.1379) effects (Richardson 2011). In order to test whether each next step significantly improved the model, robust *F*-tests (Wald tests) were performed (Hayes and Cai 2007).

## Results

### Preliminary Analyses

Table 1 presents the means and standard deviations of all variables for the total sample, and separately for boys and girls. As coercive, prosocial, and inspirational behavioral strategies, resource control and popularity were all standardized within classes (and not within the total sample), the means and standard deviations are not exactly 0 and 1, respectively.

**Table 1:** Means and standard deviations (in parentheses) of main study variables in the total sample and for boys and girls

Variable	Total	Boys	Girls	*t* (617)	df	*p*
	(*N* = 619)	(*n* = 326)	(*n* = 293)			
Coercive strategies	0.02	0.11	−0.08	2.56	617	0.011
	(0.92)	(0.95)	(0.87)			
Prosocial strategies	0.02	0.04	−0.01	0.65	617	0.514
	(0.90)	(0.93)	(0.86)			
Inspirational behavior	0.02	−0.12	0.18	−3.95	617	0.000
	(0.94)	(0.90)	(0.96)			
Social competence	2.58	2.61	2.54	1.88	617	0.061
	(0.47)	(0.47)	(0.46)			
Social manipulation	2.09	2.27	1.88	6.89	616.37	0.000
	(0.75)	(0.77)	(0.67)			
Resource control	0.02	−0.05	0.10	−2.00	613.81	0.046
	(0.94)	(1.01)	(0.84)			
Popularity	0.00	0.02	−0.03	0.65	617	0.516
	(1.00)	(1.02)	(0.98)			

Table 1 shows that boys received significantly higher ratings for coercive strategies and reported higher scores for social manipulation than girls. Girls received significantly higher ratings for inspirational behavior and resource control than boys. There were no gender differences for prosocial strategies, popularity, and social competence (the difference for social competence only approached significance).

Table 2 presents the correlations between the study variables. Coercive, prosocial, and inspirational behavioral strategies were positively correlated with each other, as well as positively correlated with both resource control and popularity. Resource control and popularity were also strongly positively correlated. Social competence displayed significant positive correlations with all variables and social manipulation was also correlated positively with all variables, except inspirational behavior. There was no significant correlation between social competence and social manipulation. Similar patterns were obtained for both genders separately, but for girls the correlations between prosocial strategies and social competence and between social manipulation and resource control were not significant. (Table 3)

**Table 2:** Correlations between main study variables (*N* = 619)

	1.	2.	3.	4.	5.	6.
1. Coercive strategies	–					
2. Prosocial strategies	0.64***	–				
3. Inspirational behavior	0.38***	0.34***	–			
4. Social competence	0.15***	0.09*	0.19***	–		
5. Social manipulation	0.16***	0.14***	0.01	0.07	–	
6. Resource control	0.55***	0.44***	0.53***	0.27***	0.12**	–
7. Popularity	0.37***	0.30***	0.45***	0.35***	0.18***	0.65***

*p < 0.05; **p < 0.01;*** *p* < 0.001

**Table 3:** Correlations between main study variables for boys (*n* = 326) and girls (*n* = 293)

		1.	2.	3.	4.	5.	6.	7.
1.	Coercive strategies	–	0.64***	0.43***	0.13*	0.13*	0.53***	0.33***
2.	Prosocial strategies	0.64***	–	0.36***	0.06	0.16**	0.44***	0.32***
3.	Inspirational behavior	0.39***	0.33***	–	0.21***	0.03	0.55***	0.47***
4.	Social competence	0.16**	0.12*	0.20***	–	−0.02	0.28***	0.33***
5.	Social manipulation	0.14**	0.13*	0.07	0.10	–	0.08	0.14*
6.	Resource control	0.58***	0.45***	0.51***	0.28***	0.19***	–	0.66***
7.	Popularity	0.40***	0.28***	0.45***	0.37***	0.20***	0.66***	–

Boys’ correlations are printed below the diagonal; girls’ correlations above the diagonal

*p < 0.05; **p ≤ 0.01; ** *p* ≤ 0.001

### Predictors of Resource Control

The combined main effects of coercive, prosocial, and inspirational behavioral strategies, social competence, social manipulation, and gender (Step 1) explained 45% of the variance in resource control (*R*^2^ = 0.452; *F*(6,612) = 96.093, *p* < 0.001). Of the total of 17 interactions added in Step 2 (strategies x social skills), Step 3 (strategies x gender and social skills x gender), and Step 4 (strategies x social skills x gender), only the interaction between coercive strategy use and social manipulation (Step 2) was significant (*β* = 0.13, SE(HC4) = 0.070, *p* = 0.009, *η*_*p*_^2^ = 0.017). However, the robust *F*-test showed that Step 2 did not significantly explain additional variance (Δ*R*^2^ = 0.010, *F*(6,606) = 1.344, *p* = 0.236), nor did Step 3 (Δ*R*^2^ = 0.004, *F*(5,601) = 1.166, *p* = 0.325) and Step 4 (Δ*R*^2^ = 0.004, *F*(6,595) = 0.724, *p* = 0.630). In order to avoid interpreting main or interaction effects that may be significant by chance, Cohen and colleagues advice to only evaluate regression coefficients at each step if the predictors in the step as a whole explain a statistically significant portion of variance in resource control at the *p* < 0.05 level (Cohen et al. 2003, p. 188). Therefore, only the standardized coefficients from Step 1 were evaluated and are reported in Table 4 (see the Appendix for the coefficients of all predictors and interactions for resource control).

**Table 4:** *R*^2^ change, standardized regression coefficients, standard errors using the HC4 estimator, *p*-values, and partial eta-squared values for the prediction of resource control of the significant steps

Predictor	Resource control				
	Δ*R*^2^	*β*	SE	*p*	*η*_*p*_^2^
Step 1	0.452			0.000	
Gender		0.094	0.060	0.004	0.014
Coercive strategies		0.338	0.047	0.000	0.102
Prosocial strategies		0.095	0.043	0.022	0.009
Inspirational behavior		0.323	0.039	0.000	0.129
Social competence		0.151	0.066	0.000	0.038
Social manipulation		0.063	0.040	0.052	0.007

Table 4 shows that gender was a significant predictor, such that girls received higher ratings on resource control than boys (small effect). All three behavioral strategies were significant positive predictors of resource control, such that those who received higher ratings for these behaviors scored higher on resource control. Partial eta-squared values indicated medium to large effects for coercive and inspirational behavioral strategies, and a small effect for prosocial strategies. Social competence and social manipulation were also positive predictors of resource control, such that those with higher scores on these skills scored higher on resource control (small effects).

### Predictors of Popularity

The same analytic strategy was used with popularity serving as the dependent variable. The main effects of the three behavioral strategies, social competence, social manipulation, and gender (Step 1) altogether accounted for 33% of the variance in popularity (*R*^2^ = 0.327; *F*(6,612) = 46.146, *p* < 0.001). None of the individual two-way interactions (Steps 2 and 3) were significant and, moreover, robust *F*-tests showed that neither Step 2 (Δ*R*^2^ = 0.012, *F*(6,606) = 1.790, *p* = 0.099), nor Step 3 (Δ*R*^2^ = 0.004, *F*(5,601) = 0.617, *p* = 0.687) explained additional variance. However, Step 4 (i.e., the three-way interactions between the behavioral strategies, social skills, and gender) explained an additional 2% of the variance (Δ*R*^2^ = 0.021, *F*(6,595) = 2.758, *p* = 0.012). In line with Cohen et al. (2003), only the standardized coefficients from Step 1 and Step 4 were evaluated and are reported in Table 5 (see the Appendix for the coefficients of all predictors and interactions for popularity).

**Table 5:** *R*^2^ change, standardized regression coefficients, standard errors using the HC4 estimator, *p*-values, and partial eta-squared values for the prediction of popularity of the significant steps 1 and 4

Predictor	Popularity				
	Δ*R*^2^	*β*	SE	*p*	*η*_*p*_^2^
Step 1	0.327			0.000	
Gender		−0.003	0.075	0.931	0.000
Coercive strategies		0.244	0.082	0.001	0.026
Prosocial strategies		−0.014	0.086	0.855	0.000
Inspirational behavior		0.347	0.067	0.000	0.062
Social competence		0.274	0.123	0.000	0.052
Social manipulation		0.110	0.064	0.023	0.009
Step 2	0.012			0.099	
Step 3	0.004			0.687	
Step 4	0.021			0.012	
Coercive strategies x social competence x gender		0.028	0.260	0.726	0.000
Prosocial strategies x social competence x gender		−0.102	0.266	0.215	0.004
Inspirational behavior x social competence x gender		−0.042	0.197	0.510	0.001
Coercive strategies x social manipulation x gender		0.245	0.163	0.000	0.023
Prosocial strategies x social manipulation x gender		−0.160	0.158	0.013	0.011
Inspirational behavior x social manipulation x gender		−0.032	0.125	0.561	0.001
Total *R*^2^	0.364			0.000	

*β*’s, SE’s, *p*’s, and *η*_*p*_^2^’s reported are from Step 4

Table 5 shows that gender was not a significant predictor of popularity. Coercive strategies (small effect) and inspirational behavior (medium effect) were significant predictors, with a higher score for these behaviors associated with a higher score for popularity. Prosocial strategies, however, were unrelated to popularity. Social competence was a significant positive predictor of popularity (small to medium effect), as was social manipulation (small effect).

**Fig. 1:**
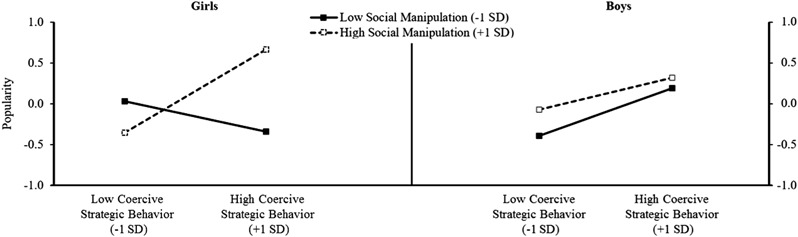
Simple slopes of coercive strategies on popularity at high and low levels of social manipulation for girls and boys

Inspection of the coefficients of Step 4 revealed two significant three-way interactions between (a) coercive strategies, social manipulation and gender (small effect), and (b) prosocial strategies, social manipulation and gender (small effect). To interpret the significant interactions, simple slope analyses were performed at the mean and one standard deviation above and below the mean of social manipulation for girls and boys (Sibley 2008). Figure 1 displays the simple slopes for coercive strategies at high and low levels of social manipulation for both girls and boys.

For girls with average social manipulation skills, the effects of coercive behavior strategies on popularity were significantly positive (*B* = 0.174, SE *B* = 0.087, *β* = 0.15, *p* = 0.047). Similarly, for girls with high levels of social manipulation skills, higher scores on coercive strategies were associated with significantly higher popularity (*B* = 0.55, SE *B* = 0.151, *β* = 0.51, *p* < 0.001). Conversely, for girls with low levels of social manipulation skills, there were no significant effects of coercive behavior strategies on popularity (*B* = −0.20, SE *B* = 0.099, *β* = −0.18, *p* = 0.039). The lower bound for the simple slope region of significance (*p* = 0.05) was −0.191 (SE = 0.097); the upper bound was 0.172 (SE = 0.088) (Preacher et al. 2010–2017).

For boys, the effects of coercive strategies on popularity at the mean level of social manipulation were significantly positive (*B* = 0.27, SE *B* = 0.082, *β* = 0.25, *p* = 0.001) and the effects of coercive strategies on popularity were significant at both high (*B* = 0.21, SE *B* = .089, *β* = 0.19, *p* = 0.017) and low (*B* = 0.32, SE *B* = 0.136, *β* = 0.29, *p* = 0.019) levels of social manipulation (see Fig. 1). So, for boys, regardless of the level of social manipulation, a higher score for coercive strategies was associated with higher popularity scores.

**Fig. 2:**
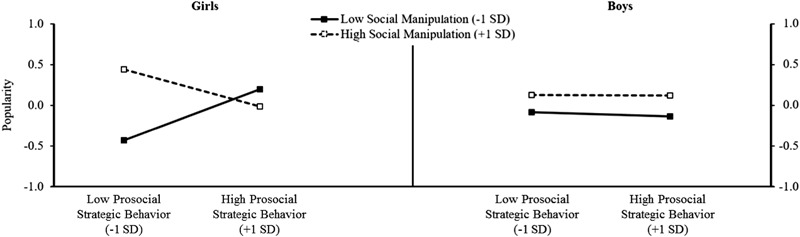
Simple slopes of prosocial strategies on popularity at high and low levels of social manipulation for girls and boys

Figure 2 displays the simple slopes for prosocial strategies at high and low levels of social manipulation for both girls and boys. Prosocial strategies were unrelated to popularity for girls with average levels of social manipulation (*B* = 0.05, SE *B* = 0.080, *β* = 0.04, *p* = 0.551), and also for girls with high levels of social manipulation (*B* = -0.24, SE *B* = 0.160, *β* = -0.22, *p* = 0.130). In contrast, for girls with low levels of social manipulation skills, higher scores on prosocial strategies were associated with significantly higher popularity (*B* = 0.33, SE *B* = 0.090, *β* = 0.30, *p* < 0.001). The lower bound for the simple slope region of significance was -0.404 (SE = 0.206); the upper bound was 0.148 (SE = 0.075). For boys, prosocial strategies were not a significant predictor of popularity regardless of level of social manipulation (*p*_s_ > 0.05).

## Discussion

Status is an important element of adolescent peer relations valued over many other domains (Brown and Larson 2009; Closson and Hymel 2016). This will be especially the case when adolescents enter a new school and establish dominance relations (Pellegrini 2007). Resource Control Theory posits that two types of behavioral strategies can be applied to gain dominance and are functionally similar: coercive strategies (i.e., instrumental aggression) and prosocial strategies (i.e., socially acceptable ways to get what you want) (Hawley 1999). These two classes of strategies pertain to two basic human motives: “getting ahead” (i.e., the need for agency) and “getting along” (i.e., the need for communion) (Hawley 2003). To the dual-motive approach, Socioanalytic Theory on personality adds a third motive: “finding meaning”. This third motive has been theoretically linked to leadership in organizations. Effective leadership would not only require aggression and prosociality, but also inspiring followers by providing meaning (Judge et al. 2009). The first goal of the present study was to investigate whether this “inspirational behavior” is also important for acquiring status and dominance in adolescence. We examined this for two forms of dominance: resource control and popularity. The second goal of this study was to examine how social skills may contribute to dominance. Dominant youth are commonly presumed to have sophisticated social skills, but empirical studies are rare. We considered both prosocial (i.e., social competence) and antisocial (i.e., social manipulation) skills and studied whether these skills were associated with dominance, either directly or by moderating (i.e., strengthening; Puckett et al. 2008; Wolters et al. 2014) the effects of the behavioral strategies. Finally, we explored whether gender qualified the relations between the behavioral strategies, social skills, and social dominance.

### Coercive, Prosocial, and Inspirational Strategies as Predictors of Resource Control and Popularity

As prosocial and inspirational strategies both refer to socially acceptable behavior, we first investigated whether the selected items indeed represented two distinct factors. In line with Socioanalytic Theory, confirmatory factor analyses indicated that the prosocial and inspirational strategy items loaded strongly on their own unique factor. Next, preliminary results showed that all three dominance-oriented strategies were positively correlated with each other and with both indicators of dominance. This is in line with the view that the strategies share a common underlying function (i.e., to yield dominance) (Hawley 2007). Finally, two hierarchical regression analyses, with also gender and social skills included as predictors, showed that both coercive and inspirational strategies were moderately strong predictors of resource control and popularity. In contrast, prosocial strategy use was a relatively weak predictor of resource control and not a significant predictor of popularity. These results showed that inspirational strategies are indeed important additional predictors of dominance in adolescence and that they are more effective for achieving dominance than prosocial strategies.

What may account for the relatively weak effect of prosocial strategies? Firstly, although studies by Hawley and colleagues found that the relatively intense use of both coercive and prosocial strategies lead to above average levels of resource control (Hawley 2007), studies by others reported that frequently and predominantly using prosocial strategies was related to only average levels of resource control in pre-adolescents (Olthof et al. 2011; Reijntjes et al. 2018). These other researchers noted that, for some items used by Hawley and colleagues, the use of a particular strategy may be confounded with the benefits that might result from using that strategy. They attempted to reword the items to avoid the items referring to both acting in a certain way and to the resources obtained by behaving in that way. Olthof et al. (2011) suggested that this approach may explain the relative weak relationship between prosocial strategies and dominance in their study and that previous research may have overestimated the dominance position that children can obtain by only or predominantly using prosocial strategies. As we used the same items as in Olthof et al., this explanation could also hold for the present results.

Secondly, although called “prosocial”, the prosocial strategies are self-serving rather than other-serving or altruistic (Hawley 2014, 2015). It is not clear to what extent prosocial strategies and prosocial behavior (i.e., caring, helping, sharing) overlap (Ostrov and Guzzo 2015), but there are indications that prosocial behavior is a positive predictor of dominance. For example, Wolters et al. (2014) found that prosocial behavior was a positive predictor of popularity in early adolescence, even when antisocial behavior (which was the strongest predictor) was taken into account. In a similar vein, Ostrov and Guzzo (2015) found that prosocial behavior in early childhood predicted resource control. This study, however, did not control for aggression. In sum, the effects of prosociality might be stronger when measured without clear self-serving benefits, but future research is needed to answer this question.

Thirdly, by including inspirational strategies, in the present study, variance in dominance previously explained by prosocial strategies may now have been accounted for by inspirational behavior, a seemingly stronger predictor of dominance. For example, sometimes an item that we would consider to tap inspirational behavior is included in the prosocial strategies scale (e.g. “Who has good ideas or suggestions that the others like to follow?”; Hawley et al. 2007). So the stronger influence of prosocial strategies in these studies may partly be due to the effect of inspirational strategies.

### Direct and Moderation Effects of Social Competence and Social Manipulation on Resource Control and Popularity

With regard to our second research goal, we expected that social competence and social manipulation would be positive predictors of resource control and popularity and would also moderate the relationships between the three behaviors and both indices of social dominance. Contrary to predictions, social competence and social manipulation did not moderate the associations between behavioral strategies and dominance. One explanation is that we investigated each behavioral strategy in isolation. Some theorists suggest that the capacity to strategically combine the use of prosocial and coercive behaviors is in itself a social skill associated with dominance (Hawley 1999; Wargo Aikens and Litwack 2011). Assessing the combined use of the three behavioral strategies simultaneously was beyond the scope of this study, but it could be the case that enhanced social skills are needed to effectively balance a range of different behaviors to gain dominance. Nevertheless, social competence and social manipulation were both positive predictors of dominance. Social manipulation was a weak predictor of both indices of dominance, whereas social competence was also a weak predictor of resource control, but a moderately strong predictor of popularity. In short, these results suggest that dominance—popularity in particular—benefits from proficiency in social skills (Judge et al. 2009) and that, as expected, both prosocial and antisocial skills matter.

### Moderation by Gender

With regard to our third research goal, we first explored whether gender moderated the main effects of the behavioral strategies and the social skills. No moderation effects were found. Next, we examined whether interactive effects between the behavioral strategies and social skills were moderated by gender. The results revealed that social manipulation moderated the association between coercive strategies and popularity, but only for girls. Specifically, using coercive strategies was associated with higher popularity for girls displaying moderate or high levels of social manipulation skills, whereas for girls with low levels of social manipulation skills, using coercive strategies was not a significant predictor of popularity.

It appears that social manipulation may be a necessary skill to be successful at using more subtle coercive strategies such as those associated with relational aggression (e.g., exclusion and rumor spreading), which is more strongly related to popularity for girls than for boys in early to middle adolescence (Rose et al. 2004). Girls are known to effectively employ gossip, spread rumors, and engage in social exclusion as a means to harm the social standing of peers, and gain and maintain their own social position (Merten 1997). This appears to be the case in the current study, as coercive strategies were only associated with popularity for girls who possessed the social manipulation skills to execute them effectively. This moderation effect was not found for boys, possibly because overtly aggressive or coercive behaviors are seen as more socially acceptable for boys (Card et al. 2008), reducing the utility of more subtle forms of coercion, and the need for social manipulation skills to execute them. This is in line with previous ethnographic studies, which have found that “toughness”, such as physical and verbal fighting skills, enhanced the likelihood of being in the top social group (Eder et al. 1995).

The results also revealed that social manipulation moderated the association between prosocial strategies and popularity, but only for girls. Unexpectedly, prosocial strategies predicted popularity only for girls who scored low on social manipulation. This may be because these girls do not possess the motivation or the necessary skills to employ coercive strategies effectively. Another explanation might pertain to the nature of the prosocial strategies. As discussed above, prosocial strategies differ from prosocial behaviors in that they are clearly self-serving. It might be that girls who score both high on socially accepted behavior clearly aimed at self-benefits and social manipulation are seen as weak or uncool. Another potential explanation is that girls who apply prosocial strategies are only viewed as popular when they score low on social manipulation and, therefore, do not evoke resistance. In any case, both moderation effects correspond with the observation that popularity depends on more subtle aspects of social interaction for girls than for boys (Cillessen 2011).

### Resource Control and Popularity as Indicators of Dominance

In line with recent views on dominance, we included both resource control and popularity as measures tapping the construct. Our results showed a strong correlation between both measures. However, slightly different patterns of predictive relations for each construct were obtained. For example, both coercive and inspirational strategies were important predictors of resource control, but inspirational strategies were most strongly associated with popularity compared to the other strategies. This may indicate that, more so than for resource control, adolescents associate popularity with individuals who display charismatic behaviors that can be admired. In short, the somewhat different patterns of predictive relationships for each construct underline the view that these measures are similar, but distinct manifestations of dominance in adolescence (Dyches and Mayeux 2015).

### Limitations and Future Directions

Like all studies, the present work has limitations. Firstly, the three-item-author-constructed inspirational scale was a first attempt to operationalize the construct. Although the items formed a reliable scale contributing considerably to the prediction of dominance, future work should examine whether the scale should be expanded. This also applies to the social manipulation scale. Secondly, our data were cross-sectional. Future research should examine the associations investigated in this study longitudinally. Such research is needed to clarify the relations among dominance, social strategies, and social skills over time, while taking gender into account. Longitudinal research may also shed more light on the factors involved in gaining versus maintaining dominance (Pellegrini et al. 2011), although clearly distinguishing between the emergence and maintenance stages of dominance may be sometimes difficult. For example, social skills involved in acquiring dominance may also play a role in maintaining it (Cillessen 2011). Thirdly, as explained in the Introduction, there are good reasons to involve both resource control and popularity as forms of dominance, but other constructs tapping peer status such as social network centrality (e.g., Freeman 1979), power (e.g., Lewis 2002), and prestige (e.g., Henrich and Gil-White 2001) are important as well. Unfortunately, both in biology and the social sciences there is no commonly accepted taxonomy of the rank ordering of individuals within groups. Many different concepts are used and the same concepts may have different meanings and ways of being operationalized, both within and across disciplines (Henrich and Gil-White 2001). With respect to inspirational behavior, the concept of prestige seems relevant. Prestige refers to freely conferred deference. Rather than using force or threats, individuals with prestige achieve status by excelling in valued domains (Henrich and Gil-White 2001). It can be hypothesized that adolescents with prestige are especially adept in using inspirational behavior effectively. Future work should examine this hypothesis. Fourthly, this study showed that social competence and social manipulation explain a significant portion of variance in resource control and popularity above and beyond coercive and inspirational strategies, but other potentially contributing skills should be investigated as well in order to determine which skills are most effective. As social skills are trainable, this would offer possibilities for interventions. Fifthly, future research may benefit from taking context into account, as the behavioral strategy used may be dependent on the type and exclusivity of the resource. For example, boys may use more aggressive strategies when competing for opposite sex contact (Pellegrini 2008), whereas for other resources, such as social contact and friendship, socially acceptable strategies may be more appropriate. Finally, our findings apply to primarily Caucasian adolescents. To examine generalizability, future studies should include youth from different ethnic backgrounds (especially non-Western; Cillessen 2011) and a broader age range (e.g., to determine at which age inspirational behavior appears).

## Conclusion

The present study adds inspirational behavior to the social tool box of coercive and prosocial strategies that adolescents can use to gain dominance, by combining two veins of research—Resource Control Theory on dominance (Hawley 1999) and Socioanalytic Theory on personality (Hogan and Blickle in press). Inspirational and coercive strategies appeared to be more effective than prosocial strategies. Next, it was shown that dominance benefits not only from proficiency in *pro*social skills (Wargo Aikens and Litwack 2011), but also from *anti*social skills. Further, gender differences regarding interactive effects between the behavioral strategies and social skills were found. They support the view that dominance as indexed by popularity depends on more subtle aspects of social interaction for adolescent girls than for boys (Cillessen 2011). Finally, slightly different patterns of predictive relations for dominance in terms of resource control and popularity were obtained. For example, compared to the other behavioral strategies, inspirational behavior was most strongly associated with popularity. This underlines the view that resource control and popularity are similar, but distinct manifestations of dominance in adolescence (Dyches and Mayeux 2015). Future research should take inspirational behavior, antisocial skills, gender differences, and different indices of dominance into account as well as examine the relations among dominance, social strategies, social skills, and gender over time. Our results highlight that adolescents striving for dominance have multiple behavioral strategies at their disposal, not only coercive and prosocial, but also the newly identified inspirational behavior.

## Appendix

Table 6

**Table 6:** *R*^2^ change, Total *R*^*2*^, standardized regression coefficients, standard errors using the HC4 estimator, *p*-values, and partial eta-squared values for the prediction of resource control and popularity of all steps

Predictor	Δ*R*^2^	Resource control				Δ*R*^2^	Popularity			
		*β*	SE	*p*	*η*_*p*_^2^		*β*	SE	*p*	*η*_*p*_^2^
Step 1	0.452			0.000		0.327			0.000	
Gender		0.080	0.067	0.024	0.010		−0.003	0.075	0.931	0.000
Coercive strategies		0.368	0.080	0.000	0.068		0.244	0.082	0.001	0.026
Prosocial strategies		0.115	0.073	0.102	0.007		−0.014	0.086	0.855	0.000
Inspirational behavior		0.367	0.070	0.000	0.081		0.347	0.067	0.000	0.062
Social competence		0.167	0.109	0.002	0.024		0.274	0.123	0.000	0.052
Social manipulation		0.075	0.066	0.159	0.005		0.110	0.064	0.023	0.009
Step 2	0.010			0.236		0.012			0.099	
Coercive x SC		−0.042	0.200	0.650	0.001		−0.116	0.192	0.163	0.006
Prosocial x SC		0.003	0.170	0.969	0.000		0.093	0.183	0.258	0.004
Inspirational x SC		0.047	0.157	0.526	0.002		0.022	0.155	0.752	0.000
Coercive x SM		0.067	0.109	0.380	0.003		−0.047	0.107	0.509	0.001
Prosocial x SM		−0.068	0.092	0.303	0.003		0.011	0.098	0.864	0.000
Inspirational x SM		−0.054	0.096	0.444	0.002		−0.043	0.085	0.455	0.001
Step 3	0.004			0.325		0.004			0.687	
Coercive x gender		−0.038	0.115	0.601	0.001		−0.055	0.120	0.447	0.001
Prosocial x gender		−0.029	0.103	0.652	0.000		0.037	0.121	0.609	0.001
Inspirational x gender		−0.042	0.088	0.508	0.001		−0.035	0.091	0.560	0.001
SC x gender		−0.016	0.140	0.734	0.000		0.006	0.169	0.908	0.000
SM x gender		−0.026	0.087	0.554	0.001		0.029	0.096	0.528	0.001
Step 4	0.004			0.630		0.021			0.012	
Coercive x SC x gender		0.032	0.253	0.705	0.000		0.028	0.260	0.726	0.000
Prosocial x SC x gender		−0.027	0.219	0.704	0.000		−0.102	0.266	0.215	0.004
Inspirational x SC x gender		−0.004	0.205	0.956	0.000		−0.042	0.197	0.510	0.001
Coercive x SM x gender		0.089	0.161	0.224	0.004		0.245	0.163	0.000	0.023
Prosocial x SM x gender		−0.019	0.140	0.755	0.000		−0.160	0.158	0.013	0.011
Inspirational x SM x gender		0.014	0.124	0.812	0.000		−0.032	0.125	0.561	0.001
Total *R*^2^	0.471			0.000		0.364			0.000	

*β*’s, SE’s, *p*’s, and *η*_*p*_^2^’s reported are from Step 4

*SC* social competence, *SM* social manipulation

## Footnotes

1 The six two-way interactions were as follows: Coercive strategies x social competence, prosocial strategies x social competence, inspirational behavior x social competence, coercive strategies x social manipulation, prosocial strategies x social manipulation, and inspirational behavior x social manipulation.

2 The five two-way interactions were as follows: Gender x coercive strategies, gender x prosocial strategies, gender x inspirational behavior, gender x social competence, and gender x social manipulation.

3 The six three-way interactions were as follows: Coercive strategies x social competence x gender, prosocial strategies x social competence x gender, inspirational behavior x social competence x gender, coercive strategies x social manipulation x gender, prosocial strategies x social manipulation x gender, inspirational behavior x social manipulation x gender.
